# Evaluation of an infrared thermography camera for measuring body temperature in dairy calves

**DOI:** 10.3168/jdsc.2022-0227

**Published:** 2022-07-09

**Authors:** M.C. Cantor, H.M. Goetz, K. Beattie, D.L. Renaud

**Affiliations:** Department of Population Medicine, University of Guelph, Guelph, ON, Canada, N1G 2W1

## Abstract

The objective of this diagnostic accuracy study was to validate an infrared thermography (IRT) camera and its software (FLIR One, FLIR, Global) for accuracy and precision for ocular temperature readings to serve as a proxy for rectal temperature in commercially housed calves. A total of 318 male Holstein calves were enrolled into this study from the day of arrival to a calf rearing facility until 14 d later. Researchers took an ocular temperature reading using an IRT camera, and a rectal temperature on each calf each day in the morning. The reference standard method for body temperature in the calves was rectal temperature. We assessed the data for agreement between the IRT and the reference standard using Pearson correlations by calf (accuracy), coefficients of determination (precision), and Bland-Altman plots for bias. In addition, a logistic regression model was built using the reference method as the outcome, with IRT as an explanatory variable to assess the diagnostic accuracy of IRT as an indicator of fever (rectal temperature ≥39.5°C). There was a negligible correlation between the IRT readings and rectal temperature (r = 0.22) and the coefficient of determination for IRT to predict rectal temperature was negligible (R^2^ = 0.05), suggesting poor precision. The average mean difference between the IRT data and rectal temperature was 0.55°C, and the differences between IRT and rectal formed a linear line around the mean difference, suggesting the Bland-Altman analyses showed proportional error and bias. The optimal probability cut-off for IRT readings for fever was at 39.5°C, and had a receiver operating characteristic area under the curve of 0.67, a sensitivity of 61%, a specificity of 71%, and 78% (3,134/4,427) of the samples were correctly labeled as either having a fever or not using IRT readings. In summary, the IRT camera and software were not validated for serving as a proxy for rectal temperature in commercially housed calves due to poor precision, and proportional error partially explained by ambient environmental conditions. We suggest that this infrared thermography system should not replace rectal temperature readings for use in commercially housed calves.

## Highlights

• An infrared thermography (IRT) camera (FLIR One Pro) was evaluated for measuring ocular temperature in calves to serve as a proxy for rectal temperature.
• Results suggested poor precision and accuracy of the IRT readings with reference to rectal temperature.
• This IRT camera cannot serve as a proxy for rectal temperature in commercially housed calves.

## Graphical Abstract

**Figure:**
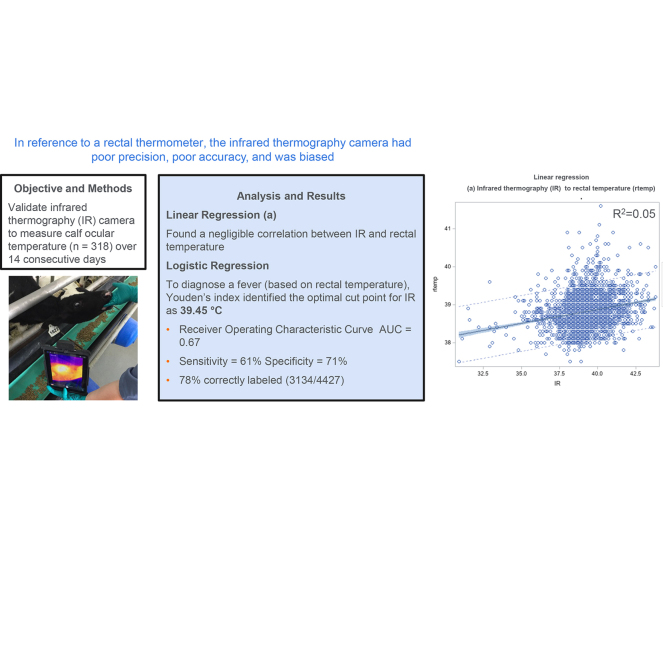
**Summary:** The objective of this study was to validate an infrared thermography (IRT) camera for measuring calf body temperature. Researchers followed a total of 318 dairy calves daily for 14 consecutive days taking ocular temperatures with IRT and rectal temperatures using a digital thermometer. With reference to the rectal thermometer, the IRT used was neither accurate nor precise, and was biased for estimating body temperature in the calves. We suggest that this IRT system cannot serve as a proxy for rectal temperature.

Infrared thermography (**IRT**) imaging has been used to document changes in physiological and metabolic status in calves (Schaefer et al., 2012; Lowe et al., 2019a,b) because an increase in radiated infrared energy is associated with a higher temperature of the object compared with other objects in the environment (Usamentiaga et al., 2014). This is important because an increased core body temperature in mammals has been associated with an immune response to an active infection (Li et al., 2020). Thus, many researchers have suggested that IRT imaging might be a useful technology to monitor calves for fever to indicate disease status, especially because IRT could be passively observed (Hoffmann et al., 2013; Lowe et al., 2020, 2021). If IRT is to be used by producers to monitor calves for disease, IRT must be precise, accurate, and without bias in comparison to rectal temperature.

Researchers have observed associations of an elevated IRT reading with disease status in calves in several locations such as the side and shoulder (Lowe et al., 2019b), the eye (Schaefer et al., 2007, 2012; Lowe et al., 2021), and most recently the cheek (Lowe et al., 2021). For example, Lowe et al. (2019b) observed that before the onset of diarrhea, shoulder temperatures decreased whereas side temperatures increased in preweaning calves serving as their own controls. Similarly, Lowe et al. (2021) observed that for the 6 d before a diarrhea bout, preweaning calves offered 5 L/d milk had decreased eye and cheek temperatures measured by IRT, though no association was observed with IRT and disease status for calves offered 10 L/d of milk. Schaefer et al. (2007) observed that in weaned beef calves, IRT ocular readings had greater positive and negative predictive values than clinical scoring at 4 to 6 d before bovine respiratory disease diagnosis. Schaefer et al. (2012) also observed that automated ocular IRT readings were higher in 200-kg steers destined for bovine respiratory disease diagnosis than healthy controls. Collectively, this research suggests that IRT readings may be useful for the automated detection of disease in calves. However, since rectal temperature has been validated to indicate fever in diseased cattle (McGuirk and Peek, 2014), it is imperative to determine if IRT readings can serve as a proxy for rectal temperature in calves.

It is important to consider the practicality of a precision technology if it is to be implemented outside of research settings. For example, several IRT calf studies validated IRT in a small sample size at one time point (Hoffmann et al., 2013, 2016), adjusted for milk intake (Bell et al., 2020; Lowe et al., 2021), or adjusted for ambient conditions such as air temperature (Scoley et al., 2019; Bell et al., 2020), humidity (Scoley et al., 2019), and wind speed (Bell et al., 2020). Based on this research, it is possible that IRT readings may be influenced by ambient environmental conditions. As IRT technology moves closer to automated collection and interpretation in calf research (Lowe et al., 2020), it is imperative to validate IRT readings in comparison to rectal temperature in calves.

The objective of this diagnostic accuracy study was to validate an IRT camera and software system (FLIR One Pro Thermal Camera, Teldyne FLIR) for accuracy and precision when measuring ocular temperature in calves in reference to rectal temperature and to quantify the diagnostic accuracy of the IRT for diagnosing calves with fever at a threshold of ≥39.5°C as determined using a rectal thermometer. This FLIR One Pro camera has an accuracy of 95% for subject temperature conditions, and we selected this camera because it would be a practical application for use by producers or veterinarians as it was developed for use with a cell phone. We hypothesized that this IRT system would be accurate and precise with reference to rectal temperature in calves.

This study was reported using the STROBE-Vet guidelines for observational studies (Sargeant et al., 2016). This prospective diagnostic accuracy study was conducted at a commercial calf raising facility in southwestern Ontario, Canada, in accordance with the University of Guelph Animal Care Committee requirements (Animal Use Protocol #4188). All calves arriving at one commercial calf rearing facility (n = 318; BW: 47.96 ± 0.22 kg) between May to August 2019 were consecutively enrolled on this study. Calves were enrolled in 4 groups based on arrival date and followed from the day of arrival for 14 consecutive days. Calves were weighed at arrival (PS2000, Brecknell Scales, Avery-Weigh Tronix) and transferred to individual pens (1.22 m^2^) with slatted floors.

A total of 300 calves were required for completion of this study. The body temperature was assumed to have normal distribution and to be within 10% of the true upper and lower population interval. The method for sample size determination described by Padgett et al. (1993) for tolerance intervals was used assuming 95% confidence in capturing 95% of the population (Padgett et al., 1993).

At 0800 h daily from arrival to d 14, an ocular temperature was taken 0.30 m from the eye of each calf and measured with a ruler using an IRT camera and software attached to one cell phone. The distance was chosen because it was the distance accessible to reach the calf eye as a trough was present in front of the pen of each calf. Simultaneously, a temperature was taken using a digital thermometer inserted into the calf's rectum. We selected ocular temperature because IRT readings taken in the calf eye have been found to be highly repeatable (Bell et al., 2020), to have high interuser reliability (Scoley et al., 2019), and to be associated with disease status in weaned beef calves (Schaefer et al., 2007, 2012), and an algorithm was recently validated to automatically process ocular IRT images in calves (Lowe et al., 2020). We took ocular IRT readings from the calves according to Lowe et al. (2019b).

A Kestrel DROP D2AG livestock heat stress monitor (Nielsen-Kellerman) was placed in the center of the calf barn approximately 2 m from the floor to record air temperature and humidity readings every 15 min. These readings were summarized into daily averages to determine if IRT was influenced by the ambient calf barn temperature-humidity index (**THI**) as adapted from NRC (2001). Furthermore, to calculate interrater Cohen's kappa and coefficients of variation for intrarater reliability, 2 IRT cameras were used to take 2 ocular IRT readings from the center of the eye of 79 calves. Simultaneously, another researcher took 2 consecutive rectal temperatures using a rectal thermometer as the reference method. Agreement was measured as whether or not the 2 readings and the IRT devices captured fever at 39.5°C.

All statistical analyses were completed using SAS version 9.4 (SAS Institute Inc.). All outliers were assessed with normality plots, histograms, and for model leverage using the residuals from a linear mixed model designed with rectal temperature as the outcome, IRT as a covariate, repeated by day, calf as the subject with unstructured covariance, and group as a random effect. The rectal thermometer was the reference method, and to evaluate agreement between the reference method and the IR camera, Pearson correlations between the IRT and rectal readings were calculated across all animals and were also calculated by calf. A linear regression was also performed to determine how much variation in rectal temperature could be explained by the IRT data. Because THI was collinear when included in the linear mixed model with IRT, a separate linear regression was performed to determine how much variance in IRT was explained by the THI data.

Precision was defined by Pearson correlations and R^2^ and was categorized according to Hinkle, 1988 (0.00–0.30 negligible, 0.30–0.50 = low, 0.50–0.70 = moderate, 0.70–0.90 = high and 0.90–1.00 = very high). We considered the IRT to be precise at measuring calf body temperature when the Pearson correlation coefficient and R^2^ were high. Bland-Altman plots (e.g., mean differences between the reference method and the IRT for calf body temperatures) were also generated as an assessment of agreement and to measure for bias; we considered the data points to be free of bias in the Bland-Altman plot if 95% of the data points were contained within the limits of agreement of the mean difference (e.g., 95% CI). We considered the IRT camera to be accurate with reference to rectal temperature in calves if the 95% interval of agreement included zero for the mean bias from the Bland-Altman plots, if the points in the Bland-Altman plot were evenly distributed around the mean difference, and if the area under the curve from the receiver operator characteristic (**ROC**) model suggested a high degree of separability (>0.90 to 1) indicating accurate classification of fever in the calves.

A ROC curve using logistic regression modeling was used to calculate the accuracy of IRT data to diagnose calves with fever as indicated by the rectal temperature readings using a threshold of ≥39.5°C (McGuirk and Peek, 2014). The optimal probability cut-off was calculated using Youden's index, which determined the threshold that maximized the sensitivity and specificity of the IRT data to detect fever in the calves.

Cohen's kappa measure of agreement for interrater reliability showed moderate agreement for the IRT readings to label fever (κ = 0.52). The coefficient of variation for temperatures within individual calf was greater for IRT readings = 1.74 than the coefficient of variation for rectal temperature = 0.73.

There were 318 calves enrolled on this study and each calf was followed for 14 consecutive days and completed this study. However, 9 observations were missing either an IRT reading or a rectal temperature reading. Thus, there were 4,443 datapoints before data cleaning. Sixteen (16/4,443) observations were filtered out of the data set and removed due to data entry error (6/16), or because of being outliers with model leverage (10/16). Thus, 318 calves with 99.6% (4,427/4,443) of the original observations were used in this validation study. The mean ± standard error ambient temperature in the calf barn was 19.65 ± 0.05°C. The mean ± standard error rectal temperature for the duration of the study using the rectal thermometer was 38.8 ± 0.001°C, whereas the mean IRT readings were 39.13 ± 0.01°C. When using a threshold of ≥39.5°C for fever diagnosis (McGuirk and Peek, 2014), 190 rectal temperature samples (4%) were positive for fever in the calves.

The Pearson correlation coefficients between the rectal temperatures determined by the reference method and the IRT readings were negligible when considering all the calves (r = 0.22; *P* < 0.001). However, when Pearson correlations were run by individual calf, the strength of the correlation between IRT and the rectal thermometer ranged from nonsignificant (n = 261/318 calves), to negligible (n = 7/318; r = 0.20; *P* < 0.10) to moderate (n = 50/318; r = 0.54, 95% CI: 0.43–0.64; *P* < 0.05; Figure 1). There was also a negligible relationship for the linear regression between the IRT and rectal thermometer readings (R^2^ = 0.05; *P* < 0.001; Figure 2A), indicating poor precision. We also observed that THI explained 25% of the variation in the IRT readings (R^2^ = 0.24; *P* < 0.001; Figure 2B). Thus, we observed poor precision between the rectal temperature data and IRT readings, though these calves were in identical environments, and it is possible that the influence of THI on IRT data may be responsible for this variation.

**Figure 1:**
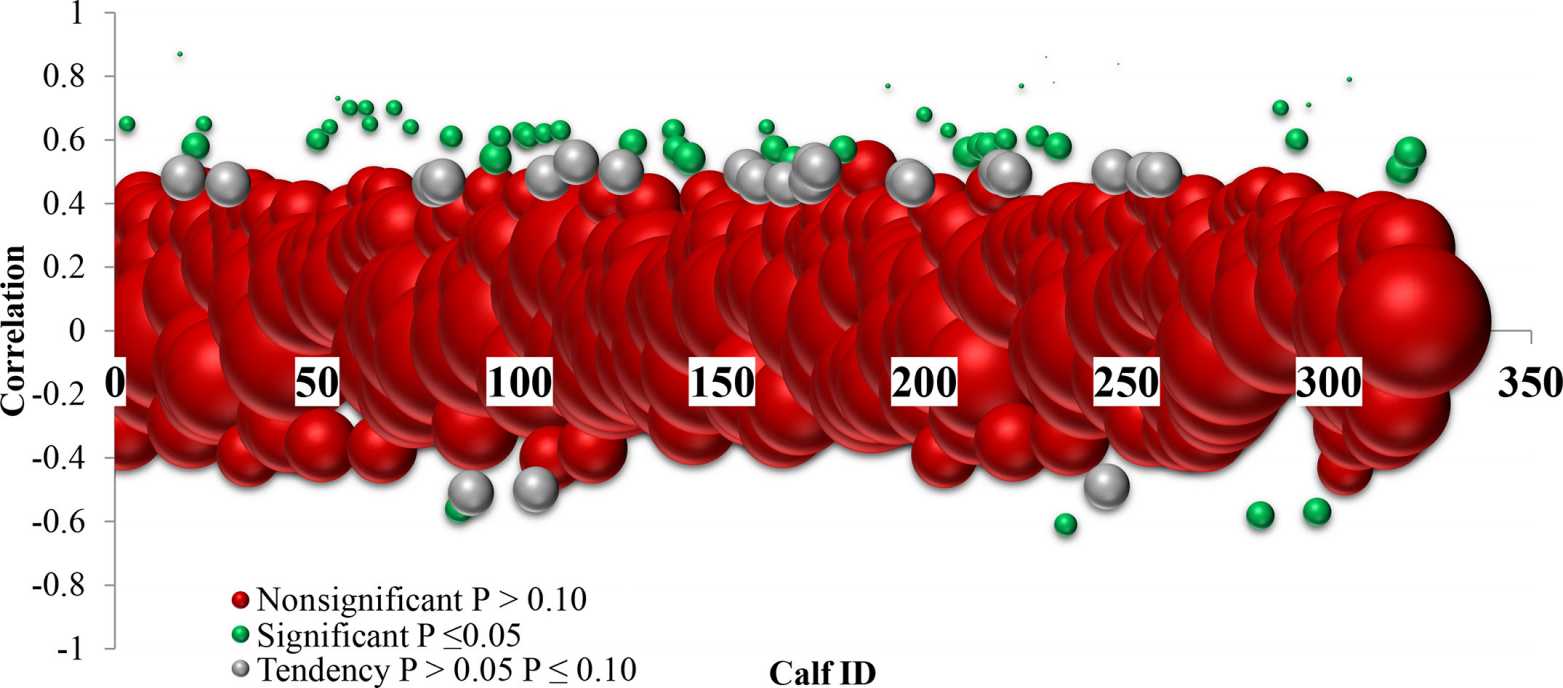
The Pearson correlation relationship between ocular temperature as measured by an infrared thermography (IRT) camera (FLIR One Pro) and rectal temperature as measured by a rectal thermometer for 4,427 temperatures taken from calves (n = 318) for 14 consecutive days. Each bubble represents a calf. Small green bubbles indicate calves (50/318) with a moderate association of IRT with rectal temperature r = 0.54 (*P-*value ≤0.05; 95% CI 0.43–0.64). Gray moderate-sized bubbles indicate calves with a tendency for an association of IRT and rectal temperature; *P-*value ≤0.10 and >0.05. Large red bubbles indicate no association (*P* > 0.10).

**Figure 2:**
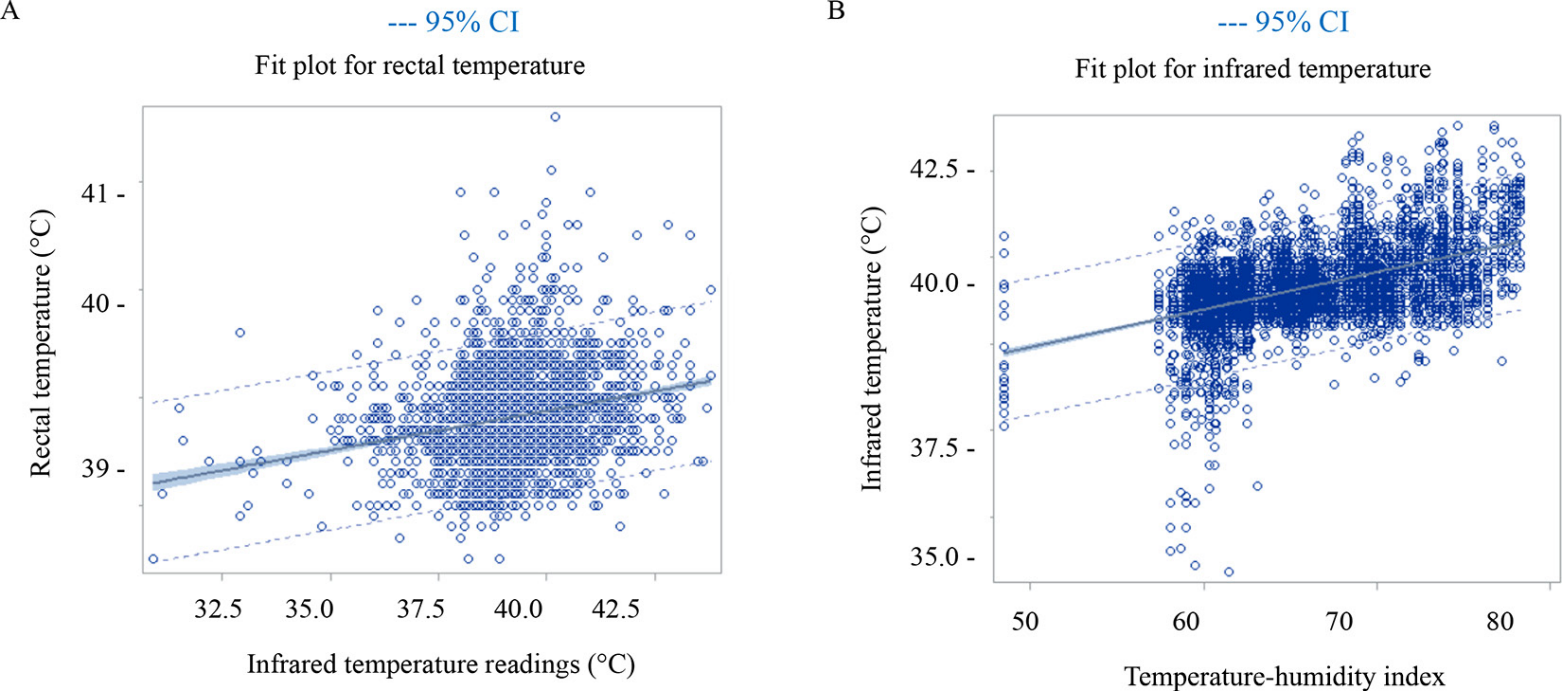
(A) A negligible linear regression (R^2^ = 0.05) demonstrating the relationship of ocular temperature readings taken by an infrared thermography camera (°C, x-axis) predicting rectal temperature (°C, y-axis) for 318 calves (4,427 observations) for 14 consecutive days. (B) A weak linear regression (R^2^ = 0.25) demonstrating the relationship of daily ambient calf barn temperature-humidity index (x-axis) predicting the infrared thermography camera readings of the calves (°C, y-axis).

A Bland-Altman plot (Figure 3) revealed that the mean bias was +0.55 ± 1.30°C and the 95% confidence interval for limits of agreement was −1.99 to 3.02°C. In addition, a visual proportional error was apparent in the Bland-Altman plot. The optimal probability cut-off for the logistic regression using Youden's index for IRT readings to diagnose fever in the calves was 39.45°C and had a ROC area under the curve of 0.67, a sensitivity of 61%, a specificity of 71%, and 71% (3,134/4,427) of the samples were correctly labeled as either positive or negative for fever using IRT readings.

**Figure 3:**
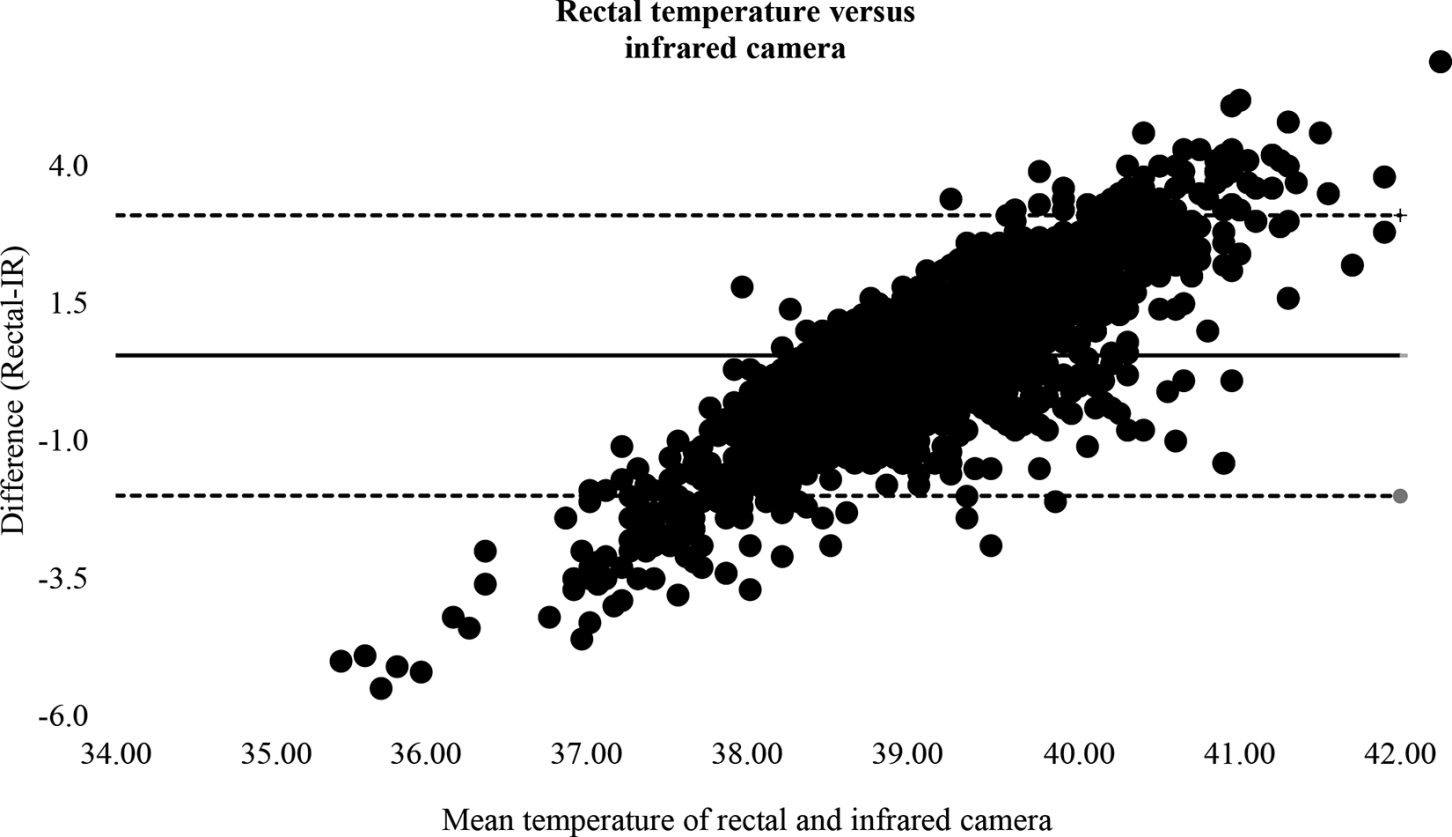
The mean differences (dotted lines represent 95% CI of the mean difference = 0.55°C) for Bland-Altman measure of agreement for calf temperature measured rectally and using an infrared thermography camera (FLIR One Pro) in 4,427 samples taken from calves (n = 318) daily for 14 consecutive days after the morning health exam.

In this study, we aimed to validate an infrared thermography camera and software for reading ocular temperature in calves in comparison to rectal temperature, and to serve as a diagnostic tool to measure fever at a threshold of ≥39.5°C for potential use on commercial calf facilities. However, in this study we observed a poor precision and negligible correlation, and a negligible amount of variation was explained between IRT readings and rectal temperature. We also observed poor accuracy for the IRT readings as the differences between IRT and rectal readings formed a linear line around the mean difference, suggesting proportional error was evident in the Bland-Altman. The optimal probability cut-off for diagnosis of fever was also not accurate enough to diagnostically replace the gold standard. We suggest that this IRT system was not validated to serve as a proxy for rectal temperature or to detect fever in calves.

Clinically, the use of this IRT camera would provide a noninvasive tool to screen calves for elevated ocular temperatures for further evaluation in a health exam. Indeed, IRT technology has been successfully validated to measure respiration rate in calves (Lowe et al., 2019a). Recently, algorithms were validated to automatically collect and interpret IRT readings of ocular and cheek temperatures in calves to look for associations with disease status (Lowe et al., 2020). However, we found that the examined IRT system cannot be used to manually scan calves for the presence of fever in reference to a rectal thermometer because the data were not precise, there was proportional error for the IRT data, and THI explained 25% of the variation in IRT readings.

The goal of this IRT system was to evaluate its use as a potential point-of-care evaluation tool to serve as a proxy for rectal temperature in calves to save time and labor. Thus, it is imperative that IRT readings are precise, accurate, and without bias, especially to justify replacement of the reference standard. We suggest that in this study, the ambient external environment influenced the relationship of IRT readings with rectal temperature. Several others have observed similar findings, that air temperature (Scoley et al., 2019; Bell et al., 2020), humidity (Scoley et al., 2019), and wind speed (Bell et al., 2020) had to be controlled for when validating IRT data for use in calves, which agreed with our findings. Indeed, researchers observed that IRT readings of an artificial cattle eye were influenced by wind speed and solar exposure, suggesting that outdoor ambient temperature may influence IRT readings in cattle (Church et al., 2014). Alternatively, it could be that characteristics of individual calves such as coat color could explain differences between calves as we observed that when correlations were performed by calf, some relationships between IRT data and rectal temperature readings were moderate, though this relationship was nonsignificant for most of the calves. Indeed, researchers have observed that IRT readings of the skin were lower in lighter colored Holsteins during the summer compared with black Holsteins (Isola et al., 2020), though this has yet to be investigated in ocular readings. Thus, we suggest IRT readings may be influenced by ambient environmental conditions and calf.

Others have validated the IRT readings to serve as a proxy for rectal temperature in calves, which disagrees with our findings. For example, ocular IRT readings and rectal temperature were found to have a moderate relationship when taken at night, but these observations were taken only at one point in time for each calf (Hoffmann et al., 2013). It is possible that because Hoffman et al. (2013) validated the IRT at night at one point in time, they did not capture the variation that we observed with this technology. Alternatively, the strength of the relationship between rectal temperature and ocular IRT in calves has been reported as weak to moderate (Hoffmann et al., 2013, 2016; Scoley et al., 2019; Bell et al., 2020). Machine learning techniques have also been used to determine if artificial intelligence of IRT readings could replace manual body temperature readings of cattle, but the coefficient of determination was weak for this algorithm as well (Ma et al., 2020). However, IRT ocular readings were validated to serve as a proxy for rectal temperature when a shed was used for the infrared thermography camera in older beef cattle (Schaefer et al., 2007, 2012). Thus, it is possible that blocking the influence of environmental factors improves the relationship between ocular IRT readings and rectal temperature in calves.

There are some limitations to consider when interpreting the results of this study. For example, we used an IRT camera, which had a 95% accuracy for subject temperature conditions because we wanted to validate a system that was practical for use by producers, researchers, and veterinarians. However, findings in our study were similar to those using a high-resolution IRT camera where poor precision was found for detecting an elevated body temperature in cattle (Ma et al., 2020). Thus, we suggest that the proportional bias observed in this study is likely responsible for the poor precision of the IRT readings in comparison to rectal temperature. However, we also observed a moderate interrater agreement for the IRT readings, and the coefficient of variation was larger compared with the rectal thermometer readings.

In conclusion, we found that IRT readings in this study were influenced by ambient conditions, and thus, this system cannot serve as a proxy for rectal temperature in commercial calf operations.
